# Hemiarthroplasty versus total hip arthroplasty for displaced femoral neck fracture in patients older than 80 years: A randomized trial protocol

**DOI:** 10.1097/MD.0000000000023530

**Published:** 2020-12-11

**Authors:** Lin Peng, Hongyu Liu, Xiaoyi Hu, Jianqiang Liu

**Affiliations:** a Department of Orthopaedics, The Fourth People's Hospital of Jinan, The Third Affiliated Hospital of Shandong First Medical University; b Department of Disease Control and Prevention, PLA 960th Hospital, Shandong, China.

**Keywords:** displaced femoral neck fracture, hemiarthroplasty, protocol, randomized controlled trial, total hip arthroplasty

## Abstract

**Background:**

The forms of treatment which are available for these patients include internal fixation, hemiarthroplasty (HA), or total hip arthroplasty (THA). Both HA and THA are widely used methods of hip replacement after displaced femoral neck fracture (DFNF). Our purpose is to analyze the long-term outcomes of these 2 different forms of treatment, which are suitable for active patients with femoral neck intracapsular fractures ≥80 years of age without advanced osteoarthritis or rheumatoid arthritis.

**Methods:**

This study is designed as a single-center randomized controlled trial. The participants will be randomly assigned to either the THA group or the HA group. Information will be collected from all participants after obtaining written informed consent in accordance with the Declaration of Helsinki and ethical board approval. Inclusion criteria include: displaced intracapsular femoral neck fracture, capability to obtain informed consent, no known metastatic disease, no contraindications to anesthesia, age ≥80 years, and ability to understand written Chinese. Patients will be evaluated at 3 months, 6 months, 1 year, and 3 years after surgery. At the time of the final follow-up, patients were assessed with use of the Harris hip score (HHS) and walking distance. Secondary outcomes of interest include postoperative complications, including 90-day medical complications (acute myocardial infarction, deep vein thrombosis, pulmonary embolism, intestinal obstruction, renal failure, and pneumonia) and surgical complications within 1 year (dislocation, infection, and revision replacement).

**Results:**

This trial is expected to be the largest randomized trial assessing the efficacy of THA and HA and powered to detect a potential difference in the primary outcome.

**Trial registration:**

This study protocol has been registered in Research Registry (researchregistry6203).

## 1 Introduction

With the aging of the world population, the proportion of elderly people is increasing, leading to an increase in the incidence of osteoporotic hip fractures.^[1]^ It is estimated that about 1.6 million hip fractures occurred in 2000, and by 2050, the incidence of hip fractures worldwide is expected to increase to >6 million.^[2–4]^ About half of hip fractures have displaced femoral neck fracture (DFNF, garden type III or IV), which may lead to nonunion or no vascular necrosis.^[5]^

The forms of treatment which are available for these patients include internal fixation, hemiarthroplasty (HA), or total hip arthroplasty (THA). Both HA and THA are widely used methods of hip replacement after DFNF. HA is a less complicated operation and is associated with lower dislocation rate, lower blood loss, and lower initial cost. However, due to complications such as acetabular erosion, some patients receiving HA treatment need to be converted to THA.^[1,6–10]^ On the other hand, THA can increase patient satisfaction and improve postoperative function. In recent years, it has been increasingly used to treat DFNF, especially in young and active emergency patients.^[11–15]^

The long-term prognosis of elderly patients (over 80 years of age) with DFNF treated with HA or THA is unclear. Several studies with a follow-up of >5 years have been published.^[16–19]^ However, the number of randomized controlled trials is small. Therefore, the treatment of these patients is still controversial, and a well conducted randomized controlled trial with large sample sizes is required. Our purpose is to analyze the long-term outcomes of these 2 different forms of treatment, which are suitable for active patients with femoral neck intracapsular fractures ≥80 years of age without advanced osteoarthritis or rheumatoid arthritis.

## 2 Material and method

### 2.1 Trial design and ethics

This study is designed as a single-center randomized controlled trial. The participants will be randomly assigned to either the THA group or the HA group. Our study protocol has been approved by the local ethics committee of the Fourth People's Hospital of Jinan Research Ethics Committee (B201010343) and has been registered in Research Registry (registration number: researchregistry6203). Information will be collected from all participants after obtaining written informed consent in accordance with the Declaration of Helsinki and ethical board approval.

### 2.2 Randomization and blinding

An author will provide a set of 280 random numbers for the allocation sequence using a website (http://www.randomization.com). The random allocation sequence will be available to this author only and thus it will be concealed from the other research team members. The included participants will be randomly assigned to either the THA group or the HA group in a ratio of 1:1. The surgeons, anesthesiologists, and nurses providing intra- and postoperative care, as well as the research coordinator assessing outcomes, are all kept blinded to allocation results.

### 2.3 Eligibility criteria

Inclusion criteria include: displaced intracapsular femoral neck fracture, capability to obtain informed consent, no known metastatic disease, no contraindications to anesthesia, age ≥80 years, and ability to understand written Chinese. The exclusion criteria are: failure to meet the inclusion criteria, including refusal of consent, radioactive osteoarthritis of hip fracture or rheumatoid arthritis; suspected pathological fractures; patients who are bedridden or could barely move to a chair; Alzheimer disease.

### 2.4 Treatment and control groups

A cemented collarless polished tapered (CPT; Zimmer, Warsaw, Indiana) femoral component is implanted in all patients via a transgluteal lateral approach. The THAs are implanted with a 28 mm cobalt chrome femoral head articulating with an all-polyethylene cemented acetabular component without a long posterior wall (ZCA; Zimmer). The HA group receives an appropriate size standard deviation femoral head internal fixator (Zimmer). The size of the head is measured from the hemispherical template during surgery and is available in 2 mm increments. Postoperatively, patients are mobilized with full weight-bearing on the second postoperative day and graduate from a walker to a cane prior to discharge.

### 2.5 Outcome measurements

Patients will be evaluated at 3 months, 6 months, 1 year, and 3 years after surgery. At the time of the final follow-up, patients were assessed with use of the Harris hip score (HHS) and walking distance. The walking distance at the time of the final follow-up is reported by the patients themselves. Anteroposterior and lateral radiographs of the involved hip are made. Secondary outcomes of interest include postoperative complications, including 90-day medical complications (acute myocardial infarction, deep vein thrombosis, pulmonary embolism, intestinal obstruction, renal failure, and pneumonia) and surgical complications within 1 year (dislocation, infection, and revision replacement).

### 2.6 Sample size calculation

The smallest difference in HHS considered to be clinically important is 10 points. In our patient population, the average HHS is estimated to be 70 points, and the maximum standard deviation is estimated to be 20 points. With an alpha level of 0.05 and a power of 90%, each group will require 83 patients. However, it is necessary to state that the 5-year expected mortality rate (average age of 84 years) for the study group is approximately 60%. Therefore, the required number has increased by 2.5 times, and each study group requires a total of 200 patients. An interim analysis of the mortality identified this to be only 41% in our selected patient group and accordingly the sample size is reduced to 140 patients per group.

### 2.7 Statistical analysis

All data will be entered into STATA software (StataCorp; Texas, USA) by 3 investigators for statistical analysis and calculation. The accuracy of these data is analyzed by 2 other researchers. According to its distribution, the Mann–Whitney *U* test or Student *t* test is used to test the statistically significant differences between the treatment groups of HHS and other continuous parameters. Chi-square test or Fisher exact test is used to analyze dichotomous variables, such as revision and dislocation rates. All analyses are 2-sided analyses, and a *P* value of <.05 is considered statistically significant.

## 3 Discussion

With the increase in the elderly population, the occurrence of femoral neck fractures becomes more and more common, which adds socio-economic significance to these fractures. Successful management is essential for each patient and the future demand for medical services. The goal of any treatment for femoral neck fractures is to restore the patient to a satisfactory functional state as quickly as possible, while minimizing morbidity and mortality, and minimizing the need for reoperation. There is ongoing debate about the comparative benefits of hemiarthroplasty and THR for patients (older than 80 years) an acute DFNF. Our purpose is to analyze the long-term outcomes of these 2 different forms of treatment, which are suitable for active patients with femoral neck intracapsular fractures ≥80 years of age without advanced osteoarthritis or rheumatoid arthritis. This trial is expected to be the largest randomized trial assessing the efficacy of THA and HA and powered to detect a potential difference in the primary outcome.

## Author contributions

**Conceptualization:** Xiaoyi Hu.

**Data curation:** Lin Peng, Hongyu Liu.

**Formal analysis:** Lin Peng, Hongyu Liu.

**Funding acquisition:** Jianqiang Liu.

**Investigation:** Lin Peng, Hongyu Liu.

**Methodology:** Xiaoyi Hu.

**Resources:** Jianqiang Liu.

**Software:** Xiaoyi Hu.

**Supervision:** Jianqiang Liu.

**Validation:** Xiaoyi Hu.

**Visualization:** Jianqiang Liu.

**Writing - original draft:** Lin Peng, Hongyu Liu.

**Writing - review & editing:** Jianqiang Liu.
